# The Application of Selenium and Copper Nanoparticles Modifies the Biochemical Responses of Tomato Plants under Stress by *Alternaria solani*

**DOI:** 10.3390/ijms20081950

**Published:** 2019-04-20

**Authors:** Tomasa Quiterio-Gutiérrez, Hortensia Ortega-Ortiz, Gregorio Cadenas-Pliego, Alma Delia Hernández-Fuentes, Alberto Sandoval-Rangel, Adalberto Benavides-Mendoza, Marcelino Cabrera-de la Fuente, Antonio Juárez-Maldonado

**Affiliations:** 1 Maestría en Ciencias en Horticultura, Universidad Autónoma Agraria Antonio Narro, Saltillo COA 25315, Mexico; t.quiterio@hotmail.com; 2 Centro de Investigación en Química Aplicada, Saltillo COA 25294, Mexico; hortensia.ortega@ciqa.edu.mx (H.O.-O.); gregorio.cadenas@ciqa.edu.mx (G.C.-P.); 3 Instituto de Ciencias Agropecuarias, Universidad Autónoma del Estado de Hidalgo, Tulancingo HID 43600, Mexico; hfad@hotmail.com; 4 Departamento de Horticultura, Universidad Autónoma Agraria Antonio Narro, Saltillo COA 25315, Mexico; asandovalr16@gmail.com (A.S.-R.); abenmen@gmail.com (A.B.-M.); cafum7@yahoo.com (M.C.-d.l.F.); 5 Departamento de Botánica, Universidad Autónoma Agraria Antonio Narro, Saltillo COA 25315, Mexico

**Keywords:** nanotechnology, biotic stress, antioxidants, defense compounds

## Abstract

Early blight is a disease that greatly affects Solanaceae, mainly damaging tomato plants, and causing significant economic losses. Although there are methods of biological control, these are very expensive and often their mode of action is slow. Due to this, there is a need to use new techniques that allow a more efficient control of pathogens. Nanotechnology is a new alternative to solve these problems, allowing the creation of new tools for the treatment of diseases in plants, as well as the control of pathogens. The aim of the present investigation was to evaluate the foliar application of selenium and copper in the form of nanoparticles in a tomato crop infested by *Alternaria solani*. The severity of *Alternaria solani*, agronomic variables of the tomato crop, and the changes in the enzymatic and non-enzymatic antioxidant compounds were evaluated. The joint application of Se and Cu nanoparticles decreases the severity of this pathogen in tomato plants. Moreover, high doses generated an induction of the activity of the enzymes superoxide dismutase, ascorbate peroxidase, glutathione peroxidase (GPX) and phenylalanine ammonia lyase in the leaves, and the enzyme GPX in the fruit. Regarding non-enzymatic compounds in the leaves, chlorophyll a, b, and totals were increased, whereas vitamin C, glutathione, phenols, and flavonoids were increased in fruits. The application of nanoparticles generated beneficial effects by increasing the enzymatic and non-enzymatic compounds and decreasing the severity of *Alternaria solani* in tomato plants.

## 1. Introduction

The tomato (*Solanum lycopersicum* L.) is the second most important cultivated horticultural crop in the world [1]. It is considered an important source of minerals, antioxidant compounds, and carotenoids such as lycopene, β-carotene and lutein, as well as other phytochemicals such as tocopherols, ascorbic acid, and polyphenols [2], so the fruit is considered a functional food that meets basic nutritional requirements. 

The world population will grow to approximately 8 billion people in 2025 and 9 billion in 2050, which has forced an increase in agricultural productivity to feed a rapidly growing world population [3]. However, food security is threatened by crop losses due to pests and attacks of pathogens, including viruses, bacteria, fungi, oomycetes, and insects [4]. It is estimated that around one third of the global crop is lost each year due to pathogenic diseases and insect injuries [5]. Early blight is a disease that significantly affects tomato plants and other plants of the family Solanaceae [6]. This disease is caused by the fungus *Alternaria solani*, which in severe cases can cause necrosis in the stem and sometimes in the fruit, while in the leaf it causes complete defoliation and later the death of the plant [7]. Chemicals such as fungicides have been used to control pests and diseases because of their economy and fast effect [5], however, they may represent environmental and health risks [8]. Although there are biological control methods, they are very expensive and slow, so they are not a good alternative. Therefore, there is a need to use new techniques for the more efficient control of pathogens without environmental risks [5]. 

In nanotechnology, innovative scientific advances have been presented, including applications in food production and agriculture [9]. Nanotechnology has the potential to make changes in the agricultural and food industry, and could be a good tool for the treatment of diseases in plants through the use of nanomaterials [10]. The nanometer scale considers dimensions of approximately at 1–100 nm and, due to this, the properties of the materials differ with respect to their physical, chemical, and biological compositions [3].

In recent years, the synthesis and use of new antimicrobial metal nanoparticles has increased due to the gradual increase in antibiotic resistance among microorganisms [11]. Nanoparticles (NPs) have been experimented with as antifungal agents against different pathogenic fungi [12]. The impact of nanoparticles on plants will depend on various parameters, such as composition, concentration, size, and the physical and chemical properties of the nanoparticles [13]. In addition, the uptake efficiency and effects of nanoparticles on growth and metabolic functions vary between plants [12]. In general, positive effects are reported in crops with the use of NPs, such as increased yield and fruit quality and increased nutrient efficiency, among others [14]. This is because NPs have a bioestimulate effect when applied in small amounts, which includes increasing tolerance to different types of stress among the observed responses [15]. However, negative effects have also been observed, such as reduction or even inhibition of growth, reduction or inhibition of germination and oxidative stress, among others [16], although this tends to happen when high doses of NPs are applied [15]. These NPs, when they reach the soil, can interact with the microorganisms that inhabit it. However, the effects are not clear, although it has been mentioned that in general nanomaterials (NMs) have a lower toxicity than their ionic forms, with the exception of those which are based on Ag [17].

Selenium nanoparticles (Se NPs) synthesized from a biological source have been shown to have antimicrobial activity against pathogenic bacteria, fungi, and yeasts [18]. They have also been shown to inhibit the growth of *Aspergillus parasiticus, A. ochraceus* and *A. nidulans* in vitro; therefore, Se NPs could be used as a fungicide in agriculture [19]. In addition, Se NPs can be less toxic to humans and animals than synthetic fungicides, which is a great advantage [19]. Further, selenium is a beneficial element for plants [20], and its application could benefit the human diet. 

Copper is an essential micronutrient that is incorporated into many proteins and enzymes, and is also important in the health and nutrition of plants [21] and in photosynthetic reactions [22]. This element, in the form of nanoparticles, has also been shown to have a strong antifungal activity [23]. It has been demonstrated that Cu NPs and Ag NPs, applied both individually and together, help in the inhibition of pathogenic fungi such as *Alternaría alternata* and *Botrytis cinera* [24]. Additionally, copper nanoparticles added to plants under conditions of abiotic stress increase the activity of antioxidant enzymes such as ascorbate peroxidase (APX), superoxide dismutase (SOD), catalase (CAT), and glutathione peroxidase (GPX) [25], which in turn can reduce oxidative stress. Experiments are commonly evaluated under controlled conditions, such as the in vitro technique, so that there is only interaction between microorganisms, or, when applications are made to plants, they are applied via the foliage or soil. Considering the above, the aim of the present investigation was to evaluate the foliar application of selenium nanoparticles and copper nanoparticles in a tomato crop infested by *Alternaria solani*, to determine the effect on growth and the production of antioxidant compounds.

## 2. Results

### 2.1. Crop Growth and Severity of Alternaria solani

The growth results of the tomato plants did not generally show significant differences with respect to *A. solani* treatment, whereas as expected, the control showed the best results in most of the variables evaluated (Table 1). The number of clusters per plant was greater with the treatment Se LD-Cu LD, surpassing the control by 8.9%. However, this result had no effect on the performance of the plants, since although this same treatment achieved the best result with *A. solani*, this were not different from the control.

**Figure 1:**
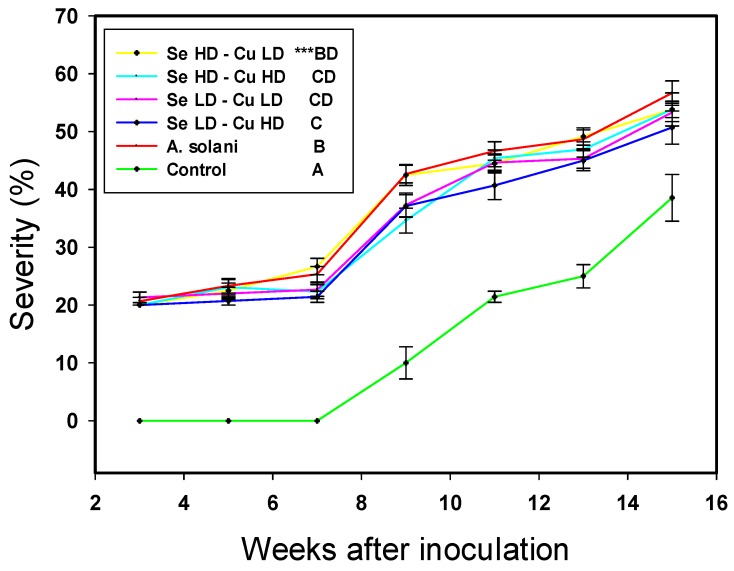
Severity of *A. solani* in tomato plants throughout crop development. Control: no inoculation of A. solani or application of NPs. A. solani: Control inoculated with A. solani without application of NPs. Se LD-Cu LD: 10 mg L^−1^ of Se NPs + 10 mg L^−1^ of Cu NPs. Se LD-Cu HD: 10 mg L^−1^ of Se NPs + 50 mg L^−1^ of Cu NPs. Se HD-Cu LD: 20 mg L^−1^ of Se NPs + 10 mg L^−1^ of Cu NPs. Se HD-Cu HD: 20 mg L^−1^ of Se NPs + 50 mg L^−1^ of Cu NPs. Bars represents the standard error of the mean. *** Significance of the multivariate analysis of variance of repeated measures (*p* < 0.0001). Different letters between treatments indicate significant differences according to the Hotteling test (*p* ≤ 0.05).

Regarding the severity of *A. solani*, the control was significantly lower than the rest of the treatments, as was expected, whereas three of the four treatments with NPs showed a significantly lower severity of *A. solani* compared to the *A. solani* treatment (Figure 1). Treatment with Se LD-Cu HD was the best in terms of decreasing the severity of *A. solani* in tomato plants over time; it consistently achieved the lowest severity compared to other *A. solani* treatments, by up to 6%. Additionally, treatments with Se LD-Cu LD and Se HD-Cu HD showed a significant decrease in the severity of *A. solani* compared to the other *A. solani* treatment, of up to 5.4%.

### 2.2. Photosynthetic Pigments

The chlorophyll content in the results was consistent; the *A. solani* treatment presented the lowest values of chlorophyll a, b and total, while the control also presented the lowest value of chlorophyll b (Table 2). The best result was observed with the Se HD-Cu LD treatment, since it presented the highest chlorophyll a, b and total values, exceeding the other *A. solani* treatments by 41, 44, and 42% respectively. The rest of the treatments with applications of NPs did not present differences with respect to the control, nor to the other treatment of *A. solani*.

### 2.3. Non-Enzymatic Compounds

The content of non-enzymatic compounds in tomato leaves was modified by the application of NPs (Table 3). However, a beneficial effect was only observed in the glutathione content where the Se HD-Cu HD treatment had the highest value, and exceeded the control by 90%. The content of vitamin C and flavonoids in the leaves was diminished with all the treatments infested with *A. solani*, regardless of whether NPs were applied. The content of phenols was not affected by any treatment.

In the case of the content of non-enzymatic compounds in the tomato fruits, the results showed that inoculation with *A. solani* was associated with the lowest values of vitamin C, glutathione, flavonoids and phenols, while the control also presented with the lowest values of vitamin C, flavonoids and phenols (Table 3). For these four compounds, at least one treatment with the application of NPs increased the content. For vitamin C, with the exception of the Se HD-Cu LD treatment, the rest of the treatments increased its content in the range of 18 to 24%. The glutathione content was better with the treatments Se HD-Cu LD and Se HD-Cu HD with respect to the treatment of *A. solani*, presenting an increase of 19 and 24%, respectively. For the content of both flavonoids and phenols, the best result was presented with the Se HD-Cu HD treatment, surpassing the control by 39 and 28%, respectively. In terms of the lycopene content in the tomato fruits, no effect was observed with any of the treatments.

### 2.4. Enzymatic Compounds

Unlike the content of non-enzymatic antioxidant compounds in tomato leaves, the enzymatic activity was modified by the application of nanoparticles (Table 4). The control showed the lowest activity of APX, GPX, SOD and phenylalanine ammonia lyase (PAL), while the treatment of A. solani was also associated with the lowest activity of APX and PAL. The treatment of Se HD-Cu HD was consistent, showing the highest activity of all the enzymes, and in which there were significant differences between treatments. APX levels surpassed both the control and *A. solani* by 438% and 436%, respectively. GPX and SOD levels only surpassed the control by 239% and 217%, respectively. The activity of PAL exceeded the control by 278%, and *A. solani* by 127%. Only the activity of CAT was not modified by the application of the treatments.

In terms of the enzymatic activity in the fruits, the only significant differences observed between treatments were for GPX, while for the rest of the enzymes all the treatments were equal to each other. The control presented the lowest activity of GPX, while the two treatments with applications of 20 mg L^−1^ of Se NPs showed the highest activity of this enzyme, surpassing the control in the range of 263–293%.

### 2.5. Total Antioxidant Capacity

The antioxidant capacity in the leaves of tomato plants increased consistently with the application of NPs, both for lipophilic and hydrophilic compounds in the two radicals used for the evaluation (ABTS and DPPH) (Table 5). When the ABTS radical was used, the hydrophilic compounds had the highest antioxidant capacity with the Se HD-Cu HD treatment, which exceeded the control by 17.5%. In the case of lipophilic compounds, the greatest antioxidant capacity was with the Se HD-Cu LD treatment, which exceeded the control by 28%. When the DPPH radical was evaluated, differences in the antioxidant capacity of only the hydrophilic compounds were observed, where again the best treatment was the Se HD-Cu HD, surpassing the control by 57%; also, the Se HD-Cu LD treatment was superior to the control by 44%. When considering the total antioxidant capacity the results are consistent; the Se HD-Cu HD treatment was the best, overcoming the control by 17.5% when the ABTS radical was used and 22.8% with the DPPH radical.

In terms of the antioxidant capacity in the tomato fruits, differences were only observed in the hydrophilic and lipophilic compounds evaluated with the ABTS radical, where in both cases the Se LD-Cu HD treatment had the lowest value in relation to the control. The total antioxidant capacity also showed the lowest value with this treatment in relation to the control, regardless of the type of radical used for the evaluation.

## 3. Discussion

### 3.1. Crop Growth and Severity of Alternaria solani

Selenium is a beneficial element for plants, although it is not essential [20], so it is possible to observe positive effects when it is applied to crops, especially if it is applied as a nanoparticle, as in the present study. Cu NPs have also shown stimulating effects on the growth of tomato crops [25]. However, in accordance with the observed results, the application of Se NPs and Cu NPs had no effect on the growth of the tomato plants, even when they were inoculated with *A. solani*. Nandini et al. [26] reported that, when applying 100 ppm of Se NPs, the growth of the plant was not affected and it did not show any phytotoxic effect, similar to the results observed here. Sathiyabama and Manikandan [27] undertook foliar applications of copper nanoparticles-chitosan, resulting in more leaves, a greater height of the stems, and more fresh weight of the plants. Although it has been reported that both Se NPs and Cu NPs can induce changes in plant growth, this is not always the case, as demonstrated in this study. 

*Alternaría solani* is the causative agent of early blight disease in tomato cultivation [28], that in severe cases can cause necrosis in the stem and sometimes in the fruit, while in the leaf it causes complete defoliation and later the death of the plant [7]. In this study, a high severity of this pathogen was not observed in the tomato plants, a fact that was confirmed by the lack of significant differences in the growth variables. However, it is unclear whether a significant decrease in severity was observed due to the application of treatments with NPs. This could be because the plants generate a series of defense mechanisms that provide protection against fungal attack [29]. Moreover, both the application of Cu NPs [30,31] and Se NPs [32,33] has been shown to be efficient in the control of pathogens in plants.

### 3.2. Photosynthetic Pigments

Chlorophyll is very important for photosynthesis of the plant, and is contained in photosystems I and II. Phytopathogenic fungi can negatively affect the photosynthetic activity of plants through the necrosis they produce in leaves due to the reduction in the content of chlorophyll [34]. Therefore, maintaining the chlorophyll content in plants when they are attacked by pathogens is vital, as this will allow the plant to continue to perform photosynthesis. The observed results showed that *A. solani* decreases the chlorophyll content; however, it could be increased with the application of Se NPs and Cu NPs, which is consistent with other scientific reports. Concentrations of 300 ppm of zinc oxide nanoparticles increased the chlorophyll a, b and total content in *Helianthus annuus* [35]. Venkatachalam et al. [36] observed the same when applying 200 mg L^-1^ of zinc oxide nanoparticles in cotton plants. Although the increase of chlorophyll observed in this work did not affect the final growth of the plant, it is possible to expect positive effects in crop cycles over a longer time frame than considered by this research.

### 3.3. Non-Enzymatic Compounds

The exposure of plants to different types of stress, whether biotic or abiotic, leads to a deregulation or interruption of the electric transport chain, which has as a consequence the overproduction of reactive oxygen species (ROS), which are strong oxidizing agents and harmful to cells [37]. The mechanism plants have to defend themselves from these ROS involves enzymatic and non-enzymatic compounds, which can eliminate ROS and thus protect cells from oxidative damage [38]. The production and accumulation of several antioxidants, such as vitamin C, phenolic acids, glutathione, carotenoids, flavonoids, is a natural response of plants against stress [39]. Different studies have confirmed that the application of nanoparticles can induce the production of these types of antioxidant compounds, which at a given moment can be beneficial to increase the tolerance against pathogens in plants. Particularly, glutathione is a non-protein thiol compound which plays an important role in the defense response of plants during biotic stress [40]. Therefore, the increase of this compound observed in this study may represent an advantage in the attack of pathogens. 

The application of nanoparticles of chitosan in leaves of *Camellia sinensis* increased the content of phenols by 24% [37]. The addition of zinc nanoparticles in potato plants increased the content of phenolic compounds [41]. In tomato plants, the application of chitosan-PVA + Cu NPs was investigated, and up to a 63% increase was observed in the content of total phenols in the leaves [25]. Although this was not observed in this work, our result may be due to the concentrations used.

The application of Cu NPs increased the content of flavonoids and glutathione in tomato fruits by 36.14% [42], coinciding with what is observed here. The application of Cu NPs + chitosan in tomato plants increased the lycopene content in fruits by 12% [43], an effect that was not observed here with the application of Se NPs and Cu NPs. However, a positive effect was clearly observed in the content of vitamin C, glutathione, flavonoids and phenols in the fruits, which, considering the studies mentioned, can be attributed to the application of NPs.

### 3.4. Enzymatic Compounds

As mentioned, the antioxidant defense system of plants also involves the participation of enzymatic compounds such as APX, GPX, CAT and SOD, which eliminate ROS [38]. It has been demonstrated that the application of Se NPs via the foliage increases the activity of SOD and CAT in sorghum plants subjected to stress, which increased the tolerance of the plants [44]. The application of Cu NPs + chitosan also increased the activity of CAT in tomato plants [43]. Likewise, the application of chitosan-PVA + Cu NPs in tomato plants increased the activity of APX and GPX [25]. Additionally, the application of NPs of zinc oxide in plants of *Vicia faba* increased the activity of the enzyme GPX [45]. These results are consistent with those obtained here, so it can be considered that tomato plants will be more able to tolerate stress under these treatments.

Phenylalanine ammonia lyase is an enzyme that participates in the defense response of plant cells in both biotic and abiotic stress [46]. This enzyme is of great importance since it is essential in the synthesis of defense metabolites against pathogens [25]. For example, the formation of phenylpropanoid phytolalexins after fungal infection implies a very rapid induction of PAL [47]. Like the antioxidant enzymes, the PAL enzyme was also increased by the application of Se NPs and Cu NPs, which may result in an increased ability to tolerate the attack of pathogens such as *A. solani*.

### 3.5. Total Antioxidant Capacity

The total antioxidant capacity of the plants is the result of the sum of all the antioxidant compounds present in these. The different types of antioxidants can be hydrophilic in nature, such as vitamin C, or lipophilic, such as β-carotene [48]. Among the most important plant compounds with antioxidant activity are carotenoids, flavonoids, and other phenolic compounds [48]. As found in this study, several authors have confirmed that the application of nanoparticles induces an increase in antioxidant capacity, probably due to the increase observed in both enzymatic and non-enzymatic compounds. Hernández-Hernández et al. [25] showed an increase in the antioxidant capacity determined by the radical ABTS when applying chitosan-PVA + Cu NPs in tomato plants. López-Vargas et al. [42], when applying 50 and 125 mg L^−1^ of Cu NPs, observed an increase in the antioxidant capacity determined by the radical ABTS.

## 4. Materials and Methods 

### 4.1. Crop Development

An indeterminate growth tomato type, saladette "El Cid F1" (Harris Moran, Davis, CA, USA), which is susceptible to *Alternaria solani*, was developed under greenhouse conditions and under a soilless cultivation system. The transplant was carried out in black polyethylene bags with a capacity of 10 L, using a mixture of peat moss and perlite as a substrate at a 1:1 ratio (*v/v*). The crop was managed on a single stem and cultural practices were applied. For the nutrition of the crop Steiner nutritive solution [49] was used through a directed irrigation system. The pH of the solution was adjusted to 6.5 with sulfuric acid. To determine the effects on crop growth the variables related to the growth of tomato plants were measured.

### 4.2. Application of Treatments

The experiment consisted of the application of selenium (Se NPs) and copper (Cu NPs) nanoparticles via foliage in different concentrations, to tomato crops inoculated with *Alternaria solani*. For this, the following treatments were used: a control without applications of NPs Se or *A. solani* (Control), a control inoculated with *A. solani* without application of NPs (*A. solani*), and the four combinations of low (LD) and high (HD) doses of Se NPs (10 and 20 mg L^−1^) with Cu NPs (10 and 50 mg L^−1^), plus stress by *A. solani*. The doses of the selected NPs were based on the previous works of Cumplido-Nájera et al. [30] and Safari et al. [50]. In the treatments with NPs, five applications of solution were made with the mentioned concentrations, with two weeks between applications, starting at 11 days after the transplant. The amount of solution applied per plant was 2.5, 5.5, 13, 17.1 and 17.1 mL per application, respectively, adding a total of 55.2 mL per plant throughout the experiment. 

**Figure 2:**
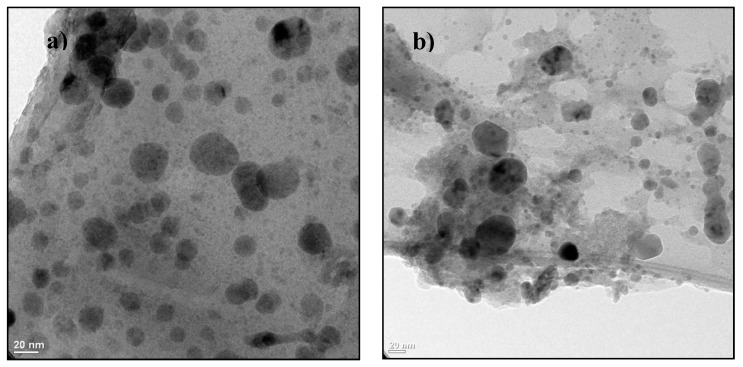
TEM micrography of selenium (**a**) and copper (**b**) nanoparticles.

The nanoparticles were synthesized in the Research Center of Applied Chemistry (Saltillo, Mexico). Se NPs were synthesized by a procedure similar to Kong et al. [51]. Using a glass reactor equipped with mechanical stirring, temperature control, and an inert atmosphere system, an aqueous solution of selenious acid (H_2_SeO_3_) and a solution of Cs-PVA were mixed at 400 rpm at a temperature of 0 °C. Subsequently, N_2_H_4_ was added to perform the reduction. The NPs are of spherical shape and size of 2–20 nm (Figure 2a). The copper nanoparticles were synthesized following the methodology described by Cadenas-Pliego et al. [52]; these NPs are spherical shape, with a size of 50 nm (Figure 2b).

### 4.3. Inoculation Preparation and Evaluation of the Severity of Alternaria solani

*Alternaría solani* was reactivated and increased in potato dextrose agar (PDA). Subsequently, they were incubated for 15 days at 27 °C [53]. The growth of the fungus, together with the PDA and sterile distilled water, was mixed, placed in a flask and stirred, then the mixture was filtered with sterile gauze and the mycelium was harvested. The liquid of all the Petri dishes was concentrated, and a spore count was made in the Neubauer chamber to adjust to a concentration of 1 × 10^6^ spores mL^−1^. Tomato plants with young and developed leaves were inoculated 28 days after the transplant (DAT) by means of a syringe infiltration technique, based on the method implemented by Smith et al. [54], and 2 mL per plant was applied. The severity scale of *Alternaría solani* was determined following the method described by Diener y Ausubel [55].

### 4.4. Sample Processing

Samples of leaf tissue were collected from randomly selected plants, and the four fully expanded young leaves were taken for biochemical analysis. One part of the samples was used to perform the determinations in fresh tissue immediately, and the other samples were stored at –20 °C and then lyophilized for 72 h at −84 °C and 0.060 mbar in a freeze dryer (Labconco, FreeZone 2.5 L model, MO, USA). The fruit samples were collected and handled as described by López-Vargas et al. [42]. For this, uniformly sized fruits were collected at stage six (light red) of maturity, according to the color visual scale of USDA [56]. The fruits were selected after verifying that they were not physically damaged and that they were uniform, and were washed. Six fruits were used whole to perform the determinations in fresh tissue immediately, and six fruits were frozen at −20 °C and then lyophilized for 72 h at −84 °C and 0.060 mbar in a freeze dryer (Labconco, FreeZone 2.5 L model, MO, USA). The lyophilized samples were ground to a fine powder to perform the determinations in lyophilized tissue.

### 4.5. Photosynthetic Pigments

The content of chlorophyll was determined according to the method of Nagata and Yamashita [57]. The absorbance at 645 y 663 nm was determined, and used in Equations (1) and (2) to determine the content of chlorophyll, as follows:

(1) $$\mathrm{Chl}\;a=0.999\times {\mathrm{Abs}}_{663}-0.0989\times {\;\mathrm{Abs}}_{645},$$

(2) $$\mathrm{hl}\;b=-0.328\times {\mathrm{Abs}}_{663}+1.77\times {\;\mathrm{Abs}}_{645},$$

The total chlorophyll is the sum of Chl a and Chl b. All data are expressed as mg 100 g^−1^ fresh weight (mg 100 g^−1^ FW).

### 4.6. Non-Enzymatic Antioxidant Compounds

For determination of phenols, the sample (0.2 g) was extracted with 1 mL of a water:acetone solution (1:1). The mixture was vortexed for 30 s. The tubes were centrifuged (Thermo Scientific Mod. ST 16R centrifuge, Langenselbold, Germany) at 17,500× *g* for 10 min at 4 °C. In a test tube, 50 μL of the supernatant, 200 μL of the Folin–Ciocalteu reagent, 500 μL of 20% sodium carbonate (Na_2_CO_3_), and 5 mL of distilled water were added and then vortexed for 30 s. The samples were placed in a water bath at 45 °C for 30 min. Finally, the reading was taken at an absorbance of 750 nm using a plastic cell in a UV-Vis spectrophotometer (Thermo Fisher Scientific, G10S model, Waltham, MA, USA). The results were expressed in equivalent milligrams of gallic acid per 100 g dry weight (mg EGA 100 g^−1^ DW).

The flavonoids quantification was carried out using Dowd’s method, adapted by Arvouet-Grand et al. [58]. For the extraction, 100 mg of lyophilized tissue was placed in a test tube, where 10 mL of reagent grade methanol was added and shaken for 30 s until the mixture was homogenized. The mixture was filtered using No. 1 Whatman paper. For the quantification, 2 mL of the extract and 2 mL of methanolic solution of aluminum trichloride (AlCl_3_) 2% were added to a test tube and left to rest for 20 min in the dark. The reading was then taken in a UV-Vis spectrophotometer (Thermo Fisher Scientific, G10S model, Waltham, MA, USA) at a wavelength of 415 nm using a quartz cell. The flavonoid content was expressed in equivalent milligrams of quercetin per 100 g of dry weight (mg EQ 100 g^−1^ DW).

The same extract was used to determine the total proteins [59], glutathione (GSH) [60], ascorbate peroxidase (APX) [61], glutathione peroxidase (GPX) [62], superoxide dismutase (SOD) (SOD Cayman 706002^®^ kit [43], and catalase (CAT) [63]. For this, 200 mg of lyophilized tissue was placed in a 2 mL Eppendorf tube. Then, 20 mg of polyvinylpyrrolidone and 1.5 mL of phosphate buffer were added.

The quantification of total proteins was determined using Bradford’s colorimetric technique [59]. In a microplate, 5 μL of the extract and 250 μL of Bradford reagent were placed in each well. They were incubated for 10 min at room temperature (26 °C), and then read at a wavelength of 630 nm on a microplate reader (BioTek, ELx808 model, Winooski, VT, USA). The total proteins were expressed in mg g^−1^ of DW.

Glutathione quantification was performed using the spectrophotometric technique by Xue et al. [60], by means of a 5,5-dithio-bis-2 nitrobenzoic acid (DTNB) reaction. A mix of 0.480 mL of the extract, 2.2 mL of sodium dibasic phosphate (Na_2_HPO_4_ at 0.32 M), and 0.32 mL of the DTNB dye (1 mM) was placed in a test tube. Then, the mix was vortexed and read on a UV-Vis spectrophotometer (Thermo Fisher Scientific, G10S model, Waltham, MA, USA) at 412 nm using a quartz cell. The results were expressed in mmol equivalent of GSH ml^−1^ min^−1^ total protein^−1^ (mmol mL^−1^ min^−1^ TP).

The content of lycopene was determined according to Nagata and Yamashita [57]. The absorbance at 453, 505, 645 y 663 nm was determined, and used in Equation (3), as follows:

(3) $$\mathrm{Lycopene}=-0.0458\times {\mathrm{Abs}}_{663}+0.204\times {\mathrm{Abs}}_{645}+0.372\times {\mathrm{Abs}}_{505}-0.0806\times {\mathrm{Abs}}_{453},$$

All data are expressed as mg 100 g^−1^ dry weight (mg 100 g^−1^ DW).

### 4.7. Enzymatic Antioxidant Compounds and Phenylalanine Ammonia Lyase

The extract used was the same as that used for total proteins. The measurement of the enzymatic activity of ascorbate peroxidase (EC 1.11.1.11) was carried out according to the method established by Nakano and Asada [61], and was expressed as U per gram of total proteins (U g^−1^ TP), where U is equal to μmol of oxidized ascorbate per milliliter per minute. The measurement was undertaken at two moments (at time 0 (T0) and at time 1 (T1)). At T0, a mix of 100 μL of extract, 500 μL of ascorbate (10 mg L^−1^), 400 μL of H_2_SO_4_ (5%), and 1 mL of H_2_O_2_ (100 mM) were placed in a test tube, and then vortexed for 30 s. The absorbance was measured in a UV-Vis spectrophotometer (Thermo Fisher Scientific, G10S model, Waltham, MA, USA) at 266 nm with a quartz cell. At T1, 100 μL of extract, 500 μL of ascorbate (10 mg L^−1^), and 1 mL of H_2_O_2_ (100 mM) were added to the previous mixture and vortexed for 1 min at a temperature of 26 °C. To stop the reaction, 400 μL of H_2_SO_4_ (5%) was added, and the absorbance was measured. Ascorbate peroxidase determination is based on the quantification of the ascorbate oxidation rate by means of the absorbance difference (T0–T1). The enzymatic activity was expressed as U per total proteins (U TP^−1^), where U is equal to μmol QE of oxidized ascorbate per milliliter per minute.

The glutathione peroxidase (QE 1.11.1.9) enzyme was determined with the method modified by Flohé and Günzler [62], and adapted by Xue et al. [60], using H_2_O_2_ as the substrate. A mix of 200 μL of extract, 400 μL of GSH (0.1 mM), and 200 μL of Na_2_HPO_4_ (0.067 M) was placed in a test tube. The mixture was preheated in a water bath at 25 °C for 5 min, then 200 μL of H_2_O_2_ (1.3 mM) was added to start the catalytic reaction for 10 min at a temperature of 26 °C. The reaction was stopped by the addition of 1 mL of 1% trichloroacetic acid. The mixture was placed in an ice bath for 30 min, and then centrifuged at 1008 *g* for 10 min at 4 °C. To assess the glutathione peroxidase, 480 μL of the supernatant, 2.2 mL of Na_2_HPO_4_ (0.32 M), and 320 μL of 5,5-dithio-bis-2-nitrobenzoic acid dye (DTNB) of 1 mM were placed in a test tube. The absorbance was measured by a UV-Vis spectrophotometer (Thermo Fisher Scientific, G10S model, Waltham, MA, USA) at 412 nm with a quartz cell. The results are expressed in U per total proteins (U TP^−1^), where U is equal to mM equivalent of GSH per milliliter per minute.

The determination of superoxide dismutase (QE 1.15.1.1) enzymatic activity was carried out using the SOD Cayman 706002^®^ kit. A mix of 20 μL of extract, 200 μL of the radical detector (tetrazolium salt), and 20 μL of xanthine oxidase solution was placed in a microplate. The microplate was covered with a transparent cover (kit), stirred for 10 s, and then incubated at 26 °C for 30 min. The absorbance was then measured at a length of 450 nm using a plate reader (BioTek, ELx808 model, Winooski, VT, USA). The principle of the test is based on the use of a tetrazolium salt for the detection of superoxide radicals generated by xanthine oxidase and hypoxanthine. One unit of SOD is defined as the amount of enzyme needed to exhibit 50% dismutation of the superoxide radical. The results are expressed in U per milliliter (U mL^−1^).

The catalase (QE 1.11.1.6) enzymatic activity was quantified by the spectrophotometric method used by Dhindsa et al. [63]. The measurement was carried out in two steps (at time 0 (T0) and at time 1 (T1)). At T0, 100 μL of extract, 400 μL of H_2_SO_4_ (5%), and 1 mL of H_2_O_2_ (100 mM) were added to an Eppendorf tube and vortexed for 30 s. The absorbance was then measured on a UV-Vis spectrophotometer (Thermo Fisher Scientific, G10S model, Waltham, MA, USA) with a quartz cell at 270 nm. At T1, 100 μL of extract and 1 mL of H_2_O_2_ (100 μL) were added and stirred for 1 min in a vortex at 26 °C. Then, 400 μL of H_2_SO_4_ (5%) was added to stop the reaction and the absorbance was measured by a UV-Vis spectrophotometer (Thermo Fisher Scientific, G10S model, Waltham, MA, USA) with a quartz cell at 270 nm. The determination of catalase is based on the quantification of the oxidation rate of H_2_O_2_ by absorbance difference (T0–T1). The values are expressed in U per total proteins (U TP^−1^), where U is equal to mM equivalent of H_2_O_2_ consumed per milliliter per minute.

The activity of phenylalanine ammonia lyase (PAL) (QE 4.3.1.5) was determined according to Sykłowska-Baranek et al. [64], and the results expressed as U per gram of total proteins (U g^−1^ TP), where U is equal to μmol equivalent of transcinnamic acid per milliliter per minute. A total of 0.1 mL of the enzymatic extract was taken, and 0.9 mL of *L*-phenylalanine (6 mM) was added. After 30 min of incubation at 40 °C, the reaction was stopped with 0.25 mL of 5 N HCl. The samples were placed in an ice bath, and 5 mL of distilled water was added. The absorbance was determined at 290 nm on a UV-Vis spectrophotometer.

### 4.8. Antioxidant Capacity

The antioxidant activity by ABTS (2,2′-azino-bis(3-ethylbenzthiazolin-6-sulfonic acid)) was determined by the spectrophotometric method of Re et al. [65], which is based on the discoloration of the ABTS radical cation. This radical was obtained from the reaction of ABTS at 7 mM with potassium persulfate at 2.45 mM (1:1) in the dark at 26 °C for 16 h, and then diluted with 20% ethanol to obtain an absorbance of 0.7 ± 0.01 at 750 nm. Afterwards, to determine antioxidant capacity in the hydrophilic compounds, phosphate buffer, 5 μL of extract, and 245 μL of the ABTS radical dilution (7 mM) were placed in a microplate and stirred for 5 s, and then allowed to stand for 7 min in darkness. The absorbance was measured by a plate reader (BioTek, ELx808 model, Winooski, VT, USA) at a wavelength of 750 nm. The blank was prepared with 250 μL of phosphate buffer (pH 7.0–7.2, 0.1 M). For the determination of the same in lipophilic compounds, extraction was carried out with a hexane:acetone solution. The results are expressed as Trolox equivalents in µmol per gram of dry weight (µmol g^−1^ DW).

The DPPH (2,2-Diphenyl-1-picrylhydrazyl) technique was performed according to the Brand-Williams et al. [66] methodology, with some modifications. The stock solution was prepared by mixing 2.5 mg of the DPPH radical with 100 mL of methanol. The absorbance of the solution was adjusted to 0.7 ± 0.02 at 515 nm using a UV-Vis spectrophotometer (Thermo Fisher Scientific, G10S model, Waltham, MA, USA). After this, for the hydrophilic compounds, 10 μL of extract obtained with phosphate buffer was taken, and 390 μL of the diluted DPPH radical was added. Methanol was used as a blank. The decrease in absorbance at 515 nm was measured after 30 min. For the determination of the same in lipophilic compounds, extraction was carried out with a hexane:acetone solution. The results are expressed as Trolox equivalents in µmol per gram of dry weight (µmol g^-1^ DW).

The total antioxidant capacity was obtained by the sum of the hydrophilic and lipophilic compounds [48].

### 4.9. Statistical Analysis

Six replicates per treatment were considered for each of the evaluated biochemical variables, in a completely random design. Variables of tomato growth and severity were assessed using 15 replicates. Each replicate was obtained from a different plant. An analysis of variance and the Fisher Least Significant Difference (LSD) mean test (*p* < 0.05) were performed to analyze the biochemical and tomato growth variables. To determine differences between treatments in severity of *A. solani,* a multivariate analysis of variance of repeated measures and Hotteling test (*p* ≤ 0.05) were performed. All statistical procedures were performed using the software Infostat 2018 (http://www.infostat.com.ar).

## 5. Conclusions

Inoculation with *Alternaria solani* in tomato plants did not affect their growth and development, however, a decrease of the severity of the pathogen of up to 6% was observed following the application of the nanoparticles used.

The application of Se NPs and Cu NPs increased the content of chlorophyll in the leaves, and likewise increased the activity of the antioxidant enzymes and the antioxidant capacity, which helped the plants better tolerate the stress caused by *A. solani*. Additionally, the content of non-enzymatic antioxidant compounds in tomato fruits, such as vitamin C, glutathione, flavonoids and phenols, was significantly increased with the application of Se NPs and Cu NPs, which improves the nutraceutical characteristics of these fruits.

## Figures and Tables

**Table 1:** Growth variables of tomato plants.

Treatment	SD (mm)	PH (cm)	NL	NC	NF	FW (kg)	AFW (g)	FAB (g)	DAB (g)
Control	13.01 a	2.30 a	29.21 a	6.86 c	63.21 ab	6.80 ab	107.92 ab	1659.1 a	233.17 a
*A. solani*	12.58 a	2.11 b	27.53 b	7.27 ab	67.00 a	7.04 a	105.71 ab	1608.2 ab	224.15 ab
Se LD-Cu LD	13.06 a	2.10 b	28.07 ab	7.47 a	68.73 a	7.05 a	102.74 ab	1656.0 a	232.41 a
Se LD-Cu HD	12.43 a	2.09 b	28.07 ab	7.21 abc	59.86 b	6.52 ab	109.10 a	1565.2 ab	207.44 ab
Se HD-Cu LD	12.42 a	2.12 b	27.08 b	7.00 bc	61.50 ab	6.07 b	99.39 b	1406.9 b	194.13 b
Se HD-Cu HD	13.18 a	2.14 b	29.23 a	7.31 ab	66.15 ab	6.75 ab	102.87 ab	1560.9 ab	217.79 ab
CV	11.33	8.2	6.09	7.35	14.65	16.17	11.5	19.19	21.56

Control: no inoculation of *A. solani* or application of NPs. *A. solani*: Control inoculated with *A. solani* without application of NPs. Se LD-Cu LD: 10 mg·L^−1^ of Se NPs + 10 mg L^−1^ of Cu NPs. Se LD-Cu HD: 10 mg·L^−1^ of Se NPs + 50 mg L^−1^ of Cu NPs. Se HD-Cu LD: 20 mg L^−1^ of Se NPs + 10 mg L^−1^ of Cu NPs. Se HD-Cu HD: 20 mg L^−1^ of Se NPs + 50 mg L^−1^ of Cu NPs. CV: Coefficient of variation. PH: Plant height. SD: Stem diameter. NL: Number of leaves. NC: Number of clusters. NF: Number of fruits. AFW: Average of fruit weight. FW: Fruit weight per plant. FAB: Fresh aerial biomass. DAB: Dry aerial biomass. Different letters per column indicate significant differences according to the Fisher LSD test (*p* < 0.05).

**Table 2:** Photosynthetic pigments in the leaves of tomato plants.

Treatment	Chlorophyll a (mg 100 g^−1^ FW)	Chlorophyll b (mg 100 g^−1^ FW)	Total Chlorophyll (mg 100 g^−1^ FW)
Control	13.01 a	2.30 a	29.21 a
*A. solani*	12.58 a	2.11 b	27.53 b
Se LD-Cu LD	13.06 a	2.10 b	28.07 ab
Se LD-Cu HD	12.43 a	2.09 b	28.07 ab
Se HD-Cu LD	12.42 a	2.12 b	27.08 b
Se HD-Cu HD	13.18 a	2.14 b	29.23 a
CV	11.33	8.2	6.09

Control: no inoculation of *A. solani* or application of NPs. *A. solani*: Control inoculated with *A. solani* without application of NPs. Se LD-Cu LD: 10 mg L^−1^ of Se NPs + 10 mg L^−1^ of Cu NPs. Se LD-Cu HD: 10 mg L^−1^ of Se NPs + 50 mg L^−1^ of Cu NPs. Se HD-Cu LD: 20 mg L^−1^ of Se NPs + 10 mg L^−1^ of Cu NPs. Se HD-Cu HD: 20 mg L^−1^ of Se NPs + 50 mg L^−1^ of Cu NPs. CV: Coefficient of variation. FW: Fresh weight. Different letters per column indicate significant differences according to the Fisher LSD test (*p* < 0.05).

**Table 3:** Non-enzymatic compounds in tomato leaves and fruits.

Organ	Treatment	Vitamin C (mg 100 g^−1^ FW)	Glutathione (mmol 100 g^−1^ DW)	Flavonoids (mg 100 g^−1^ DW)	Phenols (mg g^−1^ DW)	Lycopene (mg 100 g^−1^ DW)
Leaves	Control	167.2 a	1.47 b	62.4 a	6.65 a	Nd
	*A. solani*	124.7 bc	2.31 ab	38.3 b	7.09 a	Nd
	Se LD-Cu LD	140.8 ab	1.89 ab	42.7 b	7.91 a	Nd
	Se LD-Cu HD	105.6 c	1.49 b	40.0 b	6.53 a	Nd
	Se HD-Cu LD	137.9 b	2.25 ab	45.9 b	7.53 a	Nd
	Se HD-Cu HD	137.9 b	2.79 a	51.3 ab	7.25 b	Nd
	CV	16.64	37.79	25.30	17.82	-
Fruit	Control	16.6 b	0.36 ab	16.1 b	5.29 b	36.0 a
	*A. solani*	16.7 b	0.31 b	17.6 b	5.32 b	41.7 a
	Se LD-Cu LD	19.7 a	0.36 ab	19.2 ab	6.41 ab	38.8 a
	Se LD-Cu HD	20.7 a	036 ab	18.6 ab	6.28 ab	38.3 a
	Se HD-Cu LD	15.8 b	0.37 a	18.2 b	5.41 b	37.1 a
	Se HD-Cu HD	20.4 a	0.39 a	22.3 a	6.76 a	34.4 a
	CV	10.25	12.32	17.23	19.24	23.72

Control: no inoculation of *A. solani* or application of NPs. *A. solani*: Control inoculated with *A. solani* without application of NPs. Se LD-Cu LD: 10 mg L^−1^ of Se NPs + 10 mg L^−1^ of Cu NPs. Se LD-Cu HD: 10 mg L^−1^ of Se NPs + 50 mg L^−1^ of Cu NPs. Se HD-Cu LD: 20 mg L^−1^ of Se NPs + 10 mg L^−1^ of Cu NPs. Se HD-Cu HD: 20 mg L^−1^ of Se NPs + 50 mg L^−1^ of Cu NPs. CV: Coefficient of variation. FW: Fresh weight. DW: Dry weight. Nd: Not determined. Different letters per column indicate significant differences according to the Fisher LSD test (*p* < 0.05).

**Table 4:** Enzymatic antioxidant compounds and phenylalanine ammonia lyase in tomato leaves and fruits.

Organ	Treatment	APX (U g^−1^ TP)	GPX (U g^−1^ TP)	CAT (U g^−1^ TP)	SOD (U mL^−1^)	PAL (U g^−1^ TP)
Leaves	Control	0.26 c	10.40 b	14.97 a	1.52 a	2.62 b
	*A. solani*	0.26 c	15.12 ab	16.80 a	2.12 a	4.35 b
	Se LD-Cu LD	0.50 c	30.02 ab	36.35 a	1.57 a	7.48 ab
	Se LD-Cu HD	0.62 bc	19.99 ab	20.67 a	1.88 a	4.81 ab
	Se HD-Cu LD	1.16 ab	29.53 ab	37.27 a	2.15 a	6.88 ab
	Se HD-Cu HD	1.39 a	35.29 a	15.72 a	2.05 a	9.91 a
	CV	80.00	77.08	91.36	34.33	76.58
Fruit	Control	10.32 b	46.54 a	2.12 a	9.04 a	2.51 b
	*A. solani*	37.00 ab	113.23 a	2.09 a	19.45 a	6.38 a
	Se LD-Cu LD	34.08 ab	89.82 a	2.32 a	16.34 a	4.36 ab
	Se LD-Cu HD	35.39 ab	65.48 a	2.01 a	11.57 a	2.40 b
	Se HD-Cu LD	50.24 a	94.73 a	2.05 a	16.47 a	4.35 ab
	Se HD-Cu HD	54.37 a	66.60 a	1.69 a	14.56 a	4.11 ab
	CV	62.06	76.61	34.75	60.72	48.83

Control: no inoculation of *A. solani* or application of NPs. *A. solani*: Control inoculated with *A. solani* without application of NPs. Se LD-Cu LD: 10 mg L^−1^ of Se NPs + 10 mg L^−1^ of Cu NPs. Se LD-Cu HD: 10 mg L^−1^ of Se NPs + 50 mg L^−1^ of Cu NPs. Se HD-Cu LD: 20 mg L^−1^ of Se NPs + 10 mg L^−1^ of Cu NPs. Se HD-Cu HD: 20 mg L^−1^ of Se NPs + 50 mg L^−1^ of Cu NPs. CV: Coefficient of variation. APX: Ascorbate peroxidase. GPX: Glutathione peroxidase. CAT: Catalase. SOD: Superoxide dismutase. PAL: Phenylalanine ammonia lyase. TP: Total proteins. Different letters per column indicate significant differences according to the Fisher LSD test (*p* < 0.05).

**Table 5:** Total antioxidant capacity of tomato leaves and fruits.

Organ	Treatment	ABTS (µmol g^−1^ DW)		DPPH (µmol g^−1^ DW)		TAC ABTS (µmol g^−1^ DW)	TAC DPPH (µmol g^−1^ DW)
		H	L	H	L		
Leaves	Control	2.22 b	1.56 bc	1.44 d	2.19 a	3.78 bc	3.64 bc
	*A. solani*	2.31 ab	1.87 ab	1.87 abc	2.13 a	4.18 abc	4.00 abc
	Se LD-Cu LD	2.45 ab	1.87 ab	1.58 cd	2.12 a	4.32 a	3.69 bc
	Se LD-Cu HD	2.19 b	1.63 c	1.69 bcd	1.84 a	3.71 c	3.54 c
	Se HD-Cu LD	2.27 ab	2.00 a	2.08 ab	2.06 a	4.27 ab	4.14 ab
	Se HD-Cu HD	2.61 a	1.84 abc	2.27 a	2.20 a	4.44 a	4.47 a
	CV	13.50	15.62	18.71	15.65	10.58	11.28
Fruit	Control	3.18 a	1.45 ab	1.85 abc	2.24 ab	4.63 a	4.08 ab
	*A. solani*	2.83 ab	1.47 a	1.96 ab	2.03 b	4.29 abc	4.00 abc
	Se LD-Cu LD	3.00 ab	1.46 a	1.63 bc	2.32 a	4.46 ab	3.94 abc
	Se LD-Cu HD	2.75 b	1.29 c	1.56 c	2.07 b	4.04 c	3.63 c
	Se HD-Cu LD	2.88 ab	1.31 c	2.01 a	2.17 ab	4.19 bc	4.17 a
	Se HD-Cu HD	2.94 ab	1.32 bc	1.64 bc	2.05 b	4.26 abc	3.69 bc
	CV	11.18	8.26	16.47	8.26	7.91	8.68

Control: no inoculation of *A. solani* or application of NPs. *A. solani*: Control inoculated with *A. solani* without application of NPs. Se LD-Cu LD: 10 mg L^−1^ of Se NPs + 10 mg L^−1^ of Cu NPs. Se LD-Cu HD: 10 mg L^−1^ of Se NPs + 50 mg L^−1^ of Cu NPs. Se HD-Cu LD: 20 mg L^−1^ of Se NPs + 10 mg L^−1^ of Cu NPs. Se HD-Cu HD: 20 mg L^−1^ of Se NPs + 50 mg L^−1^ of Cu NPs.CV: Coefficient of variation. DW: Dry weight. TAC: Total antioxidant capacity. H: Hydrophilic compounds. L: Lipophilic compounds. Different letters per column indicate significant differences according to the Fisher LSD test (*p* < 0.05).
